# Ignored adult primary hypothyroidism presenting chiefly with persistent ovarian cysts: a need for increased awareness

**DOI:** 10.1186/1477-7827-9-119

**Published:** 2011-08-23

**Authors:** Jing Shu, Lili Xing, Lingyan Zhang, Suhua Fang, Hefeng Huang

**Affiliations:** 1 Department of Obstetrics & Gynecology, Sir Run Run Shaw Hospital, School of Medicine, Zhejiang University, Hangzhou 310016, China; 2 Department of Obstetrics & Gynecology, Xiasha Hospital, Hangzhou 310001, China; 3 Department of Reproductive Endocrinology, Women's Hospital, School of Medicine, Zhejiang University, Hangzhou 310006, China

## Abstract

**Background:**

Ovarian cysts are a common cause for gynecological surgery. However, some cysts are a direct result of endocrine disorders and do not require surgery. This report describes an unusual case in which persistent ovarian cysts are associated with primary hypothyroidism in a young woman. The data were collected by history-taking, physical examination, laboratory tests, ultrasound, magnetic resonance imaging and a histo-pathological study. In addition, the exons of the gene encoding the human follicle-stimulating hormone receptor were sequenced.

**Discussion:**

The patient had markedly elevated levels of thyroid-stimulating hormone and follicle-stimulating hormone and an enlarged pituitary gland. After treatment with thyroid hormone replacement, regression of the enlarged pituitary and the ovarian cysts was observed. The possible mechanisms of the pathophysiology are discussed below.

**Summary:**

It is necessary to consider hypothyroidism and other endocrine disorders in the differential diagnosis of adult patients with ovarian multiple cyst formation in order to prevent inadvertent ovarian surgery.

## Background

Ovarian cysts are a common cause for gynecological surgery. However, some cysts are a direct result of endocrine disorders and do not require surgery. Primary hypothyroidism is a common endocrine abnormality with thyroid hormone deficiency characterized by a slackening of metabolism leading to multiple system impairment. Hypothyroidism may cause reproductive endocrinology disorders as well. Occasionally, concomitant ovarian cyst formation is reported as Van Wyk and Grumbach syndrome (VWGS) in juvenile primary hypothyroidism [1], however, it is less common in adults. Failure to recognize hypothyroidism as an etiology of ovarian cysts could lead to inadvertent oophorectomy. The authors encountered an adult case, whose chief symptom was ovarian cysts, while her hypothyroid symptoms were ignored for a long time. To determine the need for endocrine evaluation in the patients with multiple ovarian cysts, we supplemented this case review by elucidating the pathophysiology and treatment of this syndrome and conducting an additional literature review.

## Case report

A 23-year-old female patient was referred to us with recurrent ovarian cysts after two previous operations on her ovaries. The patient underwent left oophorectomy due to acute abdominal pain caused by a left ovarian cyst rupture at the age of 19. However, 6 months later, cysts were detected in her right ovary, with the size increasing gradually to 11 cm × 7 cm × 7 cm. An ovarian cystectomy was performed on her right ovary when she was 22, however, the cysts reappeared soon post-operation. For further care, she consulted our gynecology department. Upon detailed inquiry, we learned that she had slight malaise for 5 years, which was relieved by rest but was ignored. She had normal menstruation after menarche at the age of 12, but experienced oligomenorrhea six months prior to the first surgery and continued to have irregular menstruation from that point on. Past medical and family history were otherwise unremarkable.

Physical examination revealed weight of 59.5 kg, height of 153 cm, and BMI of 25.4 kg/m^2 ^with normal intelligence. Her development and secondary sexual characteristics were normal. Her face was puffy with some pallor, and legs revealed trace edema. Examination of her thyroid revealed a normal size and consistency. No positive sign was found in her heart, lungs, liver, kidneys, breasts or nervous system. Pelvic examination revealed a painless palpable mass sized 6 cm × 5 cm × 5 cm in her right adnexal area.

**Figure 1:**
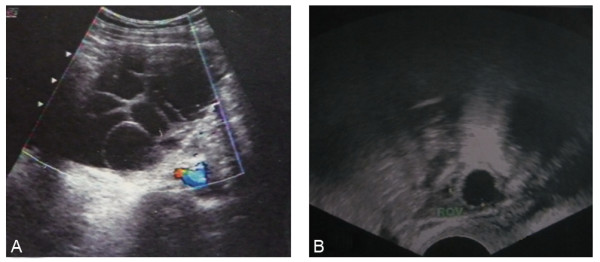
**Pelvic ultrasound image showing right ovary before (A) and after thyroxine treatment (B)**.

**Figure 2:**
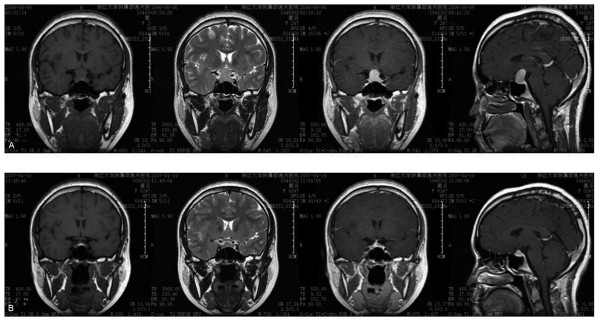
**Head MRI before (A) and after thyroxine treatment (B)**.

Initial laboratory investigations in the clinic showed normochromic anemia and unremarkable liver function tests, except for a slight rise in GST (Table 1). A lipid profile revealed dyslipidemia. A reproductive hormone test on the day of referral (66 days from the beginning of her last menstruation) showed elevated levels of FSH and PRL in addition to markedly low levels of LH and T. Abdominal ultrasound revealed mild ascites and an enlarged right ovary of 6 cm × 5 cm × 4 cm with multiple cysts divided by septa (Figure 1A). The serum level of CA-125 was normal. Considering endocrine abnormality, further examinations were performed, and the results were consistent with severe autoimmune hypothyroidism. A biochemical test detected unusually high TSH and markedly low T_3 _and T_4 _levels. Both antithyroid peroxidase and antithyroglobulin antibodies were positive. Ultrasound of the thyroid revealed both lobes to have an irregular shape with coarse texture. Electrocardiogram revealed sinus bradycardia (56 bpm). An elevated cardiac enzyme profile and cardiomegaly detected by chest x-ray revealed myocardial damage, although the patient's cardiac ejection fraction was in the normal range, as measured by echocardiogram. Brain magnetic resonance imaging (MRI) showed a compensatory hypertrophic pituitary gland, which compressed the optic chiasm and stalk (Figure 2A). However, the patient had no visual field defect. No positive findings were observed in relation to levels of corticotrophin, cortisol, immunoglobulins or other autoantibodies.

**Table 1:** Clinical information of adult hypothyroidism associated with multiple ovarian cysts

Author	Yamashita Y	Taher BM	Bassam T	Kubota K	This case
Report year	2001	2004	2006	2008	2011
Age	19	22	19	21	23
Chief presentation	Irregular cycle Weight gain	Acute abdomen	Abdomen pain	Abdomen pain	Acute abdomen Persistent cysts
Hb	106	-	-	87	87 (110-150)
TSH	132.75 (0.48-4.82)	>100 (0.47-5.01)	4191 (0.47-5.01)	1840.6 (0.5-5.5)	>100 (0.34-5.6)
FT_3_	3.08 (6.01-8.47)	-	-	-	2.00 (3.85-6.01)
FT_4_	3.86 (14.16-24.45)	<5 (9.1-23.8)	Undetectable	0 (10.42-27.41)	2.06 (7.46-21.11)
FSH	3.8	9.8	14	9.7	19.84
TPO-Ab	2.0 (0-0.3)	-	-	100	108.7 (0-5.16)
TG-Ab	-	-	-	102400	83.7 (0-4.11)
LH	1.6	12.6	1.1	<0.5	0.26
E_2_	1206	105.9	127.5	601	132
PRL	61.5 (0-26.3)	71.3	38.1 (3.8-23.2)	-	36.4
T	0.46 (0.35-2.08)	-	-	-	0 (0.35-2.43)
TG	-	-	-	-	4.40 (0.37-1.84)
TC	4.1	-	-	8.82 (3.31-5.66)	6.49 (3.10-5.69)
GPT	19	-	-	215 (6-27)	23 (5-45)
GST	14	-	-	175 (13-33)	39 (5-35)
CK	60	-	-	811 (45-163)	1148 (39-275)
LDH	340	-	-	405 (119-229)	413 (60-213)
CA125	-	93 (<35)	--	73 (1-35)	29 (<35)
Morphology of ovary	Multiple follicles R 9 cm	Multiloculated cysts R 6 cm L 5 cm	Multiple cysts R 4.5 cm L 5 cm	Multiple cysts R 10 cm L 4 cm	Multicystic R 6 cm

Hb: hemoglobin (g/l); TSH: thyroid stimulating hormone (mU/l); FT_3_: free triiodothyronine (pmol/l); FT_4_: free tetraiodothyronine (pmol/l); TPO-Ab: antithyroid peroxidase antibody (IU/ml); TG-Ab: antithyroglobulin (IU/ml); FSH: follicle-stimulating hormone (IU/l); LH: luteinizing hormone (IU/l); E_2_: estradiol (pmol/l); PRL: prolactin (ng/ml); T: testosterone (nmol/l); TG: total triglyceride (mmol/l); TC: total cholesterol (mmol/l); GPT: glutamate pyruvate transaminase (IU/l); GST: glutamic-oxaloacetic transaminase (IU/l); CK: creatine kinase (IU/l); LDH: lactate dehydrogenase(IU/l); CA-125: cancer antigen 125(IU/l); R: right ovary; L: left ovary. Data in parentheses are within the normal range.

**Figure 3:**
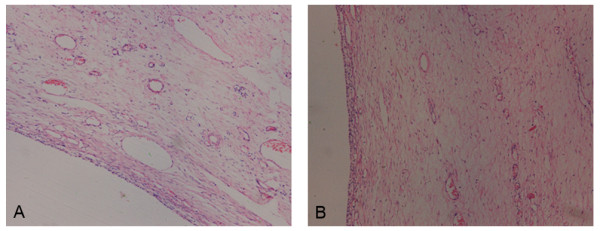
**The ovarian cyst is lined by a simple layer of non-luteinized cells (A) with edematous stroma (B)**. (HE, original magnification 100×).

On review of her previous medical records, several signs associated with a diagnosis of hypothyroidism were found, suggesting a long history of hypothyroidism. These included mild anemia (Hgb 9.7 g/dl) when she underwent the first surgery, and mildly elevated levels of GST (39 IU/l), TG (2.34 mmol/l) and LDH (254 IU/l) at the time of her second preoperative check. Histological examinations of the two previous ovarian specimens were reviewed, and both were confirmed to be follicular cysts by the presence of granulosa cells lining the cystic wall, without luteinization. The stroma was obviously edematous, but the mycoprotein stain was negative (Figure 3).

Taken together, this data suggested a diagnosis of primary hypothyroidism associated with the ovarian follicular cysts. Thyroid replacement was initiated (25 μg per day with weekly 12.5-25 μg increase to a maximum 100 μg daily). The patient's symptoms improved promptly. Within 2 months, she became biochemically euthyroid and her menstruation returned to normal. Within 4 months, the level of FSH dropped to 3.48 IU/l in the early follicular stage, and the concentrations of LH, PRL and E_2 _were 1.92 IU/l, 29.32 ng/ml and 338 pmol/l, respectively. Within 7 months, the right ovarian enlargement regressed completely (Figure 1B), and the repeat MRI of the pituitary (Figure 2B) showed dramatic shrinkage. No surgical procedures were performed. However, the patient suffered from infertility two years later. She responded poorly to ovarian stimulation when she underwent in vitro fertilization, suggesting her ovarian reserve had been destroyed.

DNA sequencing of FSH receptor exons did not reveal any mutations. The two common polymorphisms were homozygous for the Thr allele at position 307 and homozygous for the Asn allele at position 680. (Additional file 1, Figure S1; Additional file 2, Supplemental Methods).

## Discussion

### Diagnosis

Ovarian cysts are a common cause for gynecological surgery. Owing to the complexity of ovarian composition and function, the etiology of ovarian cysts can vary greatly, including benign or malignant tumors, endometriosis and inflammation, etc. Some cysts only result from reproductive endocrine dysfunction and may resolve without surgery after endocrine correction. Ovarian hyperstimulation is such a condition. With multiple follicles developing, the ultrasound images of the ovaries appear similar to multilocular cystadenoma with multiple septa. Usually, ovarian hyperstimulation syndrome (OHSS) is caused by iatrogenic superovulation. Excessive exogenous FSH stimulates multiple follicular growth simultaneously. In some rare cases, spontaneous OHSS related with pregnancy has been described as depending on activating mutations of the FSH receptor (FSHR) gene, causing ovarian hyper-responsiveness to circulating FSH or even cross-responsiveness of FSHR to hormones having a structure similar to FSH, such as human chorionic gonadotrophin (hCG) or TSH [2,3]. Eutopic or ectopic gonadotrophin adenoma secreting FSH can also present with multiple follicular cysts in ovaries [4,5]. Without considering these endocrine disorders as a possible etiology, clinicians are likely to assume a diagnosis of neoplasm, leading to unnecessary ovarian surgery.

Hypothyroidism is another endocrine disorder associated with ovarian hyperstimulation, yet is often ignored in its evaluation. There have been spontaneous OHSS cases reported in pregnant women with hypothyroidism [6-8]. In an animal model of hypothyroidism induced with thiouracil, animals exhibited an increased sensitivity to the cyst-formative action of pregnant mare serum gonadotrophin and hCG [9]. However, without hCG, hypothyroidism rarely results in the formation of enlarged multicystic ovaries. Since Van Wyk and Grumbach first described the combination of multicystic ovaries, juvenile hypothyroidism and precocious puberty in 1960, sporadic cases of this syndrome have been reported in pre-pubertal or adolescent girls [10-13]. For adults, only single-digit cases have been reported, from age 19 to 26 [14-17]. These cases are similar to the case presented here. The ultrasound pictures in each of these cases revealed similar typical multiple cysts of varied sizes, as would be expected in OHSS (Table 1).

Just as hypothyroidism may be missed as a cause of ovarian hyperstimulation, the presence of ovarian cysts are often overlooked in patients with hypothyroidism, particularly in nongestational adults. Without symptoms of precocious puberty such as the Van Wyk and Grumbach syndrome in young girls, it is much easier to ignore reproductive organ ultrasound evaluations in hypothyroid adults. Furthermore, without hCG triggering the release of certain ovarian vasoactive substances [18], these nongestational adults lack the typical complications of ovarian hyperstimulation, such as severe abdominal distention, serious hemoconcentration, massive ascites or pleural effusion. In the few such cases that have been reported, patients were referred to hospitals only due to too a large abdominal mass or acute abdomen.

### Pathophysiology

There are several hypotheses about the mechanism of ovarian cyst formation associated with hypothyroidism.

First, TSH, FSH and their receptors have related structures. Extremely high concentrations of TSH in hypothyroidism may be sufficient to cause the activation of FSHR. Experiments from Anasti validated that recombinant human TSH could elicit a dose-dependent cyclic adenosine monophosphate response in the in vitro human FSHR bioassay, though the concentration of TSH required for half-maximal stimulation was several logs greater than that of FSH [19]. This provides a major potential mechanism for severe hypothyroidism resulting in ovarian hyperstimulation.

A second possible mechanism may be related to a change in pituitary gonadotropin levels. Though gonadotropin levels of hypothyroid women are usually normal [20], the patients with multilocular ovaries reported by us and most other authors showed relatively high FSH and low LH levels. The mechanism of such endocrine change remains enigmatic. It is suggested that an "overlap" effect in the negative feedback response occurs, so that not only TSH but also gonadotropins are stimulated by extremely high TRH [1]. These remarkably high endogenous FSH levels can effectively stimulate the follicles. One reason for low LH levels despite elevated FSH may be that in the pituitary-hypothalamic axis LH and FSH synthesis and secretion are differentially regulated. Slow GnRH pulse frequencies favor FSH production and secretion, whereas rapid frequencies favor LH production and secretion [21-23]. It may be that hypothyroidism is associated with slow GnRH pulse. Blunted or delayed LH response to GnRH has been found in some hypothyroid women [20]. Another possible reason for the FSH and LH mismatch is that high TRH can induce hyperprolactinemia which also can impair LH pulse by intermittent reductions in GnRH secretion [24]. A third explanation may be the presence of another regulation pathway for FSH secretion, involving estrogen and activin at the pituitary level. In our case, due to the low level of LH, the theca cells could not produce enough testosterone for granulosa cells to biosynthesize estrogen. The low estrogen status in turn enhanced FSH secretion, persistently stimulating follicular enlargment without a concomitant rise in estrogen concentrations.

A third possible mechanism has been hypothesized to be due to FSHR activating mutations permitting or amplifying the affect of hCG or TSH on the follicles. The mutations of Asp^567^Asn, Thr^449^Ile, Thr^449^Ala and Ile^545^Thr were found in women with spontaneous OHSS in pregnancy [25-27]. FSHR gene polymorphisms may influence FSHR protein responsiveness as well, particularly codons 307 and 680 [27]. However, Ryan sequenced the FSHR gene in eight juvenile patients with VW syndrome. No FSHR mutation was identified and no difference in the responses to rFSH or rTSH in different FSHR allelic combinations was found [12]. Likewise, in our patient, we found no FSHR exon mutations. The genotype in our case was homozygous Thr^307^-Asn^680^. While FSHR gene mutations and polymorphism may be associated with ovarian hyperstimulation, there is no evidence that they are essential to its pathogenesis.

Fourth, TSH may sensitize the ovaries to gonadotropin stimulation by stimulating nuclear thyroid receptors in the granulosa cells, thereby exacerbating ovarian hyperstimulation[28].

Fifth, myxedematous-type infiltration might also account for the interference of steroidogenesis in the ovary and contribute to ovarian cystic changes.

Despite multiple hypotheses for the association between hypothyroidism and ovarian hyperstimulation, the exact mechanism is not yet clear. It is uncertain why hypothyroidism is so common while concomitant ovarian hyperstimulation appears to be so rare. All reported cases are associated with severe hypothyroidism with TSH more than 100 mU/l, and even as high as 4,191 mU/l. Besides, different pathways may function in different cases. In our case, extremely high TSH and FSH were the main causes. Furthermore, it is not fully understood why this syndrome is only seen in girls or young women. Maybe young gonads are particularly susceptible to stimulation by TSH or FSH.

### Treatment

Although the precise etiopathology of this disorder remains speculative, the treatment approach is for ovarian hyperstimulation due to hypothyroidism is clear. Evidence exists that supplementation with thyroid hormone can lead to the complete regression of the multicystic ovarian enlargement, even in patients with high CA-125 levels[15]. Surgical exploration in these cases should be performed only for emergent cases such as ovarian torsion or rupture. If at the time of surgery ovarian neoplasm is suspected, precautionary frozen sectioning should be performed prior to oophorectomy. When adequate thyroid replacement therapy fails to resolve ovarian enlargement, surgical excision should be considered. Panico reported an ovarian wedge resection for persistent ovarian enlargement after adequate thyroid replacement therapy for 14 months. The histological section in their case showed a benign ovarian cyst with extensive hemorrhage and myxedematous infiltration[10]. Other treatments, such as aspiration[11], may be considered as well for persistent cysts.

## Summary

Although the syndrome is very rare, profound hypothyroidism can cause multicystic ovaries in an adult. It is imperative that all health care providers, including gynecologists, surgeons and physicians should consider hypothyroidism and other endocrine disorders in the differential diagnosis of adult females presenting with multicystic ovarian tumors to avoid unnecessary and catastrophic ovarian resection.

## List of abbreviations

VWGS: Van Wyk and Grumbach syndrome; Hb: hemoglobin; TSH: thyroid stimulating hormone; FT_3_: free triiodothyronine; FT_4_: free tetraiodothyronine; TPO-Ab: antithyroid peroxidase antibody; TG-Ab: antithyroglobulin antibody; FSH: follicle-stimulating hormone; LH: luteinizing hormone; E_2_: estradiol; PRL: prolactin; T: testosterone; TG: total triglyceride; TC: total cholesterol; GPT: glutamate pyruvate transaminase; GST: glutamic-oxaloacetic transaminase; CK: creatine kinase; LDH: lactate dehydrogenase; CA-125: cancer antigen 125; R: right ovary; L: left ovary; MRI: magnetic resonance imaging; hCG: human chorionic gonadotrophin; OHSS: ovarian hyperstimulation syndrome; GnRH: gonadotropin releasing hormone; FSHR: follicle-stimulating hormone receptor; Thr: Threonine; Ala: Alanine; Asn: Aspatagine; Ser: Serine.

## Competing interests

The authors declare that they have no competing interests.

## Authors' contributions

JS collected the clinical information and drafted the manuscript. LX carried out the PCR and RFLP. LZ participated in data collection and the statistical analysis. SF participated in the clinical diagnosis and treatment. HH conceived of the report, and helped to draft the manuscript. All authors read and approved the final manuscript.

## Supplementary Material

Additonal file 1 **Supplemental figure S1**. RFLP analysis and sequence showing the Thr^307^Ala polymorphism. Click here for file

Additonal file 2 **Supplemental methods**. Method for RFLP analysis. Click here for file
