# Supplementary material for: Ignored adult primary hypothyroidism presenting chiefly with persistent ovarian cysts: a need for increased awareness
Source: Reprod Biol Endocrinol. 2011 Aug 23;9:119. doi: 10.1186/1477-7827-9-119 (PMC3184057; doi:10.1186/1477-7827-9-119)
Supplement: Additonal file 1 — Supplemental figure S1. RFLP analysis and sequence showing the Thr307Ala polymorphism. [file 1477-7827-9-119-S1.PDF]

# Additional file 1, Supplemental Figure S1

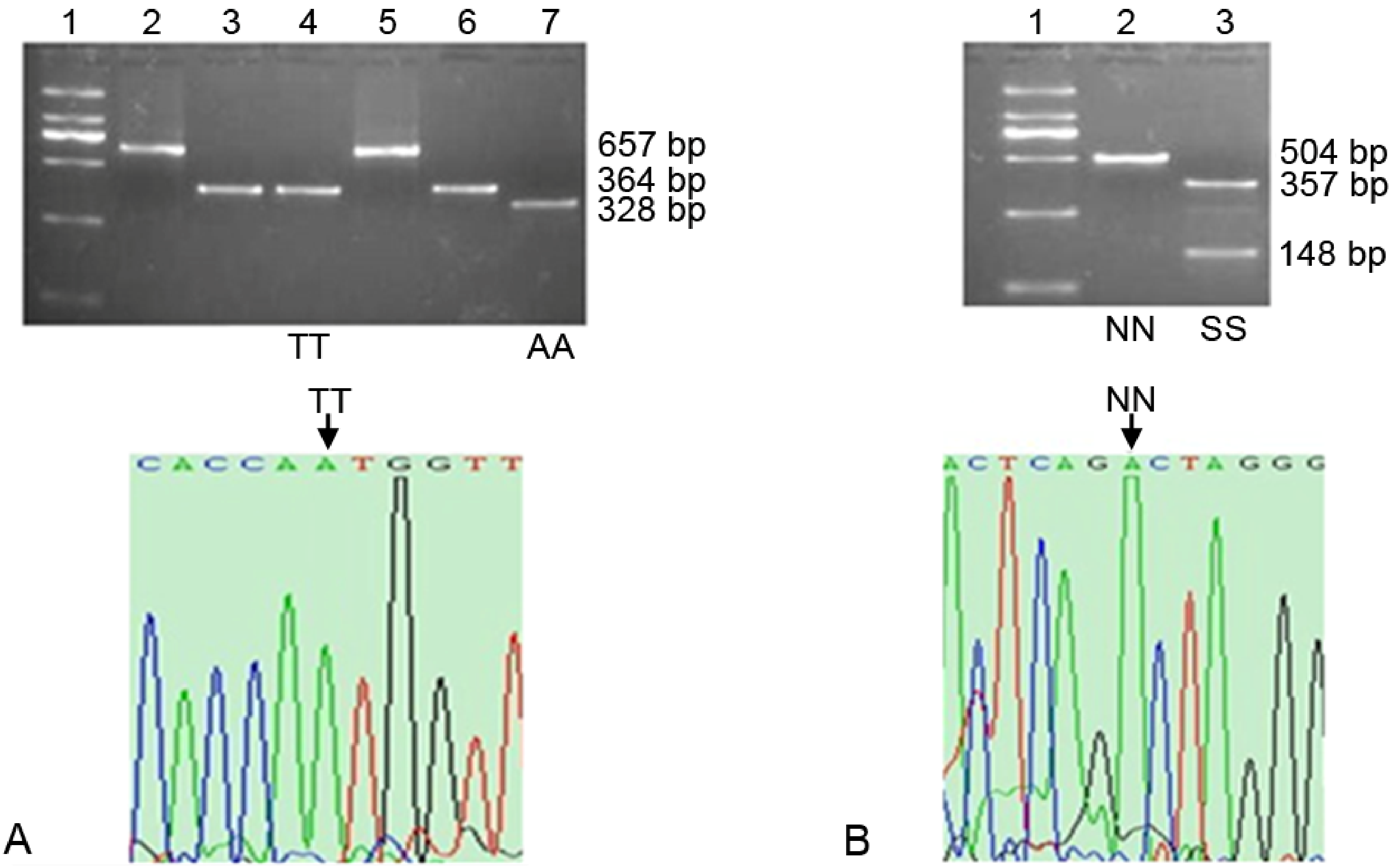


Legend A:
Lane 1: marker
Lane 2: the patient homozygous for TT, PCR product of external PCR (657 bp)
Lane 3: the patient homozygous for TT, PCR product using nested primers (364 bp)
Lane 4: digested PCR products showing that the patient is homozygous for TT (364 bp)
Lane 5: control homozygous AA PCR product of external PCR (657 bp)
Lane 6: control homozygous AA PCR product obtained using nested primers (364 bp)
Lane 7: digested PCR products showing control homozygous AA (328 bp); the 36-bp band has run off the gel
Sequence picture of the patient showing TT homozygosity.

Legend B:
Description: RFLP analysis and sequence showing the Asn680Ser polymorphism.
Lane 1: marker
Lane 2: digested PCR products showing that the patient is homozygous for NN (504 bp)
Lane 3: Digested PCR products showing that the control is homozygous for SS (357 bp and 148 bp)
Sequence picture of the patient showing NN homozygosity.
