# Supplementary material for: Ignored adult primary hypothyroidism presenting chiefly with persistent ovarian cysts: a need for increased awareness
Source: Reprod Biol Endocrinol. 2011 Aug 23;9:119. doi: 10.1186/1477-7827-9-119 (PMC3184057; doi:10.1186/1477-7827-9-119)
Supplement: Additonal file 2 — Supplemental methods. Method for RFLP analysis. [file 1477-7827-9-119-S2.PDF]

Additional file 2, Supplemental methods

Method for RFLP analysis (Sudo S, Kudo M, Wada S, Sato O, Hsueh AJ, Fujimoto S: Genetic and functional analyses of polymorphisms in the human FSH receptor gene. Mol Hum Reprod 2002, 8:893-899.)

RFLP analysis of the Thr^{307}Ala variant

Detection of the Thr^{307}Ala variant was performed using the nested PCR–RFLP method. First, primers (primer-1: 5'-TCTGAGCTTCATCCAATTTGCA-3'; primer-2: 5'-GGGAAAGAGGGCA GCTGCAA-3') amplified the 657-bp DNA fragment. The PCR product was further amplified by a second PCR using a new set of primers (primer-3: 5'-CAAATCTATTTTAAGGCAAGAAGTTGATTATATGCCTCAG-3'; primer-4: 5'-GTAGATTCCAATGCAGAGATCA-3'). In primer-3, a mismatch nucleotide has been induced. This mismatch and the A to G transition create a *Bsu36I* restriction site so that the second PCR fragment following *Bsu36I* digestion reveals different patterns. A 364-bp band for TT and a 328-bp band for AA (the 36-bp band has run off the gel).

RFLP analysis of the Asn^{680}Ser variant

The region of the FSH receptor gene of interest was amplified by PCR using genomic DNA as a template and a set of primers (primer-1: 5'- ACATCGTGTCCTCCTCTAGTG -3'; primer-2: 5'-AATGTGTAGAAGCACTGTCAGC -3') that amplified the 504-bp DNA fragment. Because the A to G transition creates an endonuclease *BsrI* recognition site, the PCR fragment reveals different patterns following *BsrI* digestion and 2.5% agarose gel electrophoresis. A 504-bp band for NN; two bands, 357 and 148 bp, for SS.
